# No silver bullet? Snow leopard prey selection in Mt. Kangchenjunga, Nepal

**DOI:** 10.1002/ece3.8279

**Published:** 2021-11-01

**Authors:** Kamal Thapa, Natalie Schmitt, Narendra M. B. Pradhan, Hem Raj Acharya, Santosh Rayamajhi

**Affiliations:** ^1^ Tribhuvan University, Institute of Forestry Kritipur Nepal; ^2^ Department of Biochemistry and Biomedical Sciences McMaster University Hamilton Ontario Canada; ^3^ International Union for the Conservation of Nature (IUCN) Kathmandu Nepal; ^4^ Department of National Parks and Wildlife Conservation Kathmandu Nepal

**Keywords:** blue sheep, common leopard, fecal, genetic analysis, snow leopard, wolf, yak

## Abstract

In this study, we investigated the impact of domestic and wild prey availability on snow leopard prey preference in the Kangchenjunga Conservation Area of eastern Nepal—a region where small domestic livestock are absent and small wild ungulate prey are present. We took a comprehensive approach that combined fecal genetic sampling, macro‐ and microscopic analyses of snow leopard diets, and direct observation of blue sheep and livestock in the KCA. Out of the collected 88 putative snow leopard scat samples from 140 transects (290 km) in 27 (4 × 4 km^2^) sampling grid cells, 73 (83%) were confirmed to be from snow leopard. The genetic analysis accounted for 19 individual snow leopards (10 males and 9 females), with a mean population size estimate of 24 (95% CI: 19–29) and an average density of 3.9 snow leopards/100 km^2^ within 609 km^2^. The total available prey biomass of blue sheep and yak was estimated at 355,236 kg (505 kg yak/km^2^ and 78 kg blue sheep/km^2^). From the available prey biomass, we estimated snow leopards consumed 7% annually, which comprised wild prey (49%), domestic livestock (45%), and 6% unidentified items. The estimated 47,736 kg blue sheep biomass gives a snow leopard‐to‐blue sheep ratio of 1:59 on a weight basis. The high preference of snow leopard to domestic livestock appears to be influenced by a much smaller available biomass of wild prey than in other regions of Nepal (e.g., 78 kg/km^2^ in the KCA compared with a range of 200–300 kg/km^2^ in other regions of Nepal). Along with livestock insurance scheme improvement, there needs to be a focus on improved livestock guarding, predator‐proof corrals as well as engaging and educating local people to be citizen scientists on the importance of snow leopard conservation, involving them in long‐term monitoring programs and promotion of ecotourism.

## Graphical abstract

The genetic analysis accounted for 19 individual snow leopards (10 male and 9 female) with a mean population size estimate of 24 (95% CI: 19–29), and an average density of 3.9 snow leopards/100 km^2^ within 609 km^2^. From the available prey biomass, we estimated snow leopards consumed 7% annually which was comprised of wild prey (49%), domestic livestock (45%), and 6% unidentified items. The estimated 47,736 kg Blue sheep biomass gives a snow leopard‐Blue sheep ratio of 1: 59 on a weight basis. The proportion of young Blue sheep was estimated at 17%, with an almost double predation rate at 28%.

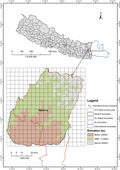

## 1 INTRODUCTION

The snow leopard (*Panthera uncia*) inhabits the mountain ranges of 12 countries across Central and South Asia, perhaps the most iconic feline of high Asia (Sharma & Singh, 2020). The global population of snow leopards is roughly estimated at fewer than 6400 individuals (Sharma & Singh, 2020) and declining due to a reduction in prey availability (Jackson et al., 2008) and killing by humans. Nowell et al. (2016) estimated that people kill 221–450 individual snow leopards annually, with 55% of these being retaliatory killings over livestock depredation. Declining prey populations is a serious and lasting risk to snow leopard survival, with the cascading effect of intensifying livestock depredation rates, inciting herder retribution (Jackson & Lama, 2016). Bagchi et al. (2019) suggested that the revival of wild prey could be one of the potential solutions for reducing livestock depredation through effective conservation intervention. However, even with an abundance of wild prey, snow leopards often target livestock as they are abundant and easy to prey on (Johansson et al., 2015; Suryawanshi et al., 2017). Khanal et al. (2020) suggested that the intensity of livestock depredation might be largely regulated by the abundance of snow leopards and availability of livestock, where depredation rates can vary, despite wild prey density levels. Not surprisingly, such predation dynamics can be temporal and highly site‐specific, with losses varying greatly between successive years and even nearby settlements (Jackson & Lama, 2016). Local communities may therefore unintentionally play an important role in sustaining snow leopard populations through their livestock, regardless of the presence or absence of wild ungulate prey. Therefore, with frequent livestock depredation, retaliatory killing of snow leopards by herders are becoming more common globally (Jackson & Lama, 2016; Lovari & Mishra, 2016), and practical solutions are becoming more challenging (Ale et al., 2016; DNPWC, 2017).

In Nepal, where snow leopards live in three main complexes along the north border of the country (western, central, and eastern), we see substantial variation and complexity in the way livestock and wild prey impact livestock depredation by snow leopards, with research heavily focused on the central complex. In the Shey Phoksundo National Park (SPNP) in midwestern Nepal, both small wild prey, comprising mostly blue sheep—*Pseudois nayaur* (2.3/km^2^) and Himalayan marmot—*Marmota himalayana* (132.6/km^2^), and small livestock (primarily sheep and goats) are available, with wild prey making up 70% of the snow leopard diet and livestock only 30% (Devkota et al., 2013). In central Nepal, in both the Manang and Nar Phu valleys, despite an abundance of small wild ungulate prey (blue sheep: 6.6–10.2 blue sheep/km^2^; Oli et al., 1993 and 8.4 blue sheep/km^2^; Thapa et al., 2021; Wegge et al., 2012), livestock (mostly small livestock breeds) comprised 35% and 42% of snow leopard diet, respectively, with wild prey (blue sheep) comprising 51.6% and 53%, respectively. Similarly, in the Annapurna–Manaslu region, Chetri et al. (2017) found that snow leopards targeted both blue sheep (7.41/km^2^, comprising 57% of their diet) and small livestock such as goats and horses that were available (27% of their diet), avoiding yak. In contrast, in eastern Nepal, within the Mt. Everest region, where small livestock are absent and much larger livestock such as yak are abundant, snow leopards were found to target mostly young Himalayan tahr (*Hemitragus jemlahicus*) to the degree of population suppression (48% of their diet), musk deer (20% of their diet), and cattle (16%–30% of their diet; Lovari et al., 2009). The results from these studies bring into question the role that livestock play in snow leopard prey preference in terms of availability and catchability, and the interplay with wild prey. A comprehensive understanding of these predator–prey dynamics is essential to formulating effective conservation strategies for snow leopards as well as assisting communities with adequate schemes to compensate livestock loss.

In the Kangchenjunga Conservation Area (KCA) of eastern Nepal, Nepal's first conservation area managed by local communities, we had the opportunity to assess snow leopard prey preference in the absence of small domestic livestock such as goats and sheep and in the presence of small wild prey such as blue sheep. Gurung et al. (2011) reported that snow leopard numbers have actually increased in this area and snow leopard killings have gone down from 1–3/year to 0/year, possibly due to the successful implementation of community‐managed livestock insurance schemes, but snow leopard prey preference could also be playing a role here. In this study, we explore the interplay between snow leopard abundance, wild prey availability, and the presence of larger livestock, such as yak, on snow leopard prey preference in the KCA. Our objectives were to (1) estimate snow leopard abundance using noninvasive genetic analysis, (2) assess the availability of wild prey, and (3) compare the presence of wild prey and domestic livestock in the snow leopard diet by macro‐ and microscopic analyses. Based on our findings, we discuss the implications for the conservation of this vulnerable species in eastern Nepal.

## 2 MATERIALS AND METHODS

### 2.1 Study area

**FIGURE 1:**
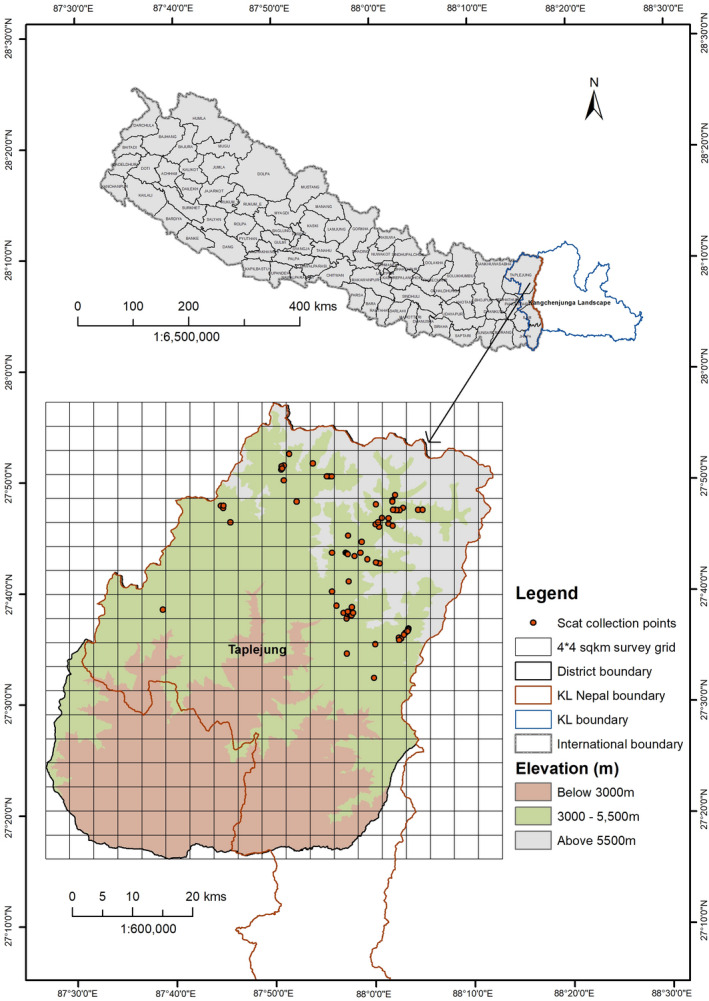
Study area location within prime snow leopard habitat in the Kangchenjunga Conservation Area, eastern Nepal, where we conducted snow leopard scat collection and blue sheep and livestock surveys during the Spring (April–May) 2012. Red dots denote putative snow leopard scat collection sites in the sampling grids (4 × 4 km^2^)

The KCA is part of the Taplejung district located in the far northeastern part of Nepal. Bordering the state of Sikkim in India to the east and the Tibet Autonomous Region (TAR) of China to the north, the district (20°24′–27°56′N & 87°39′–88°12′E) covers mid‐hills to high mountain terrains including Mt. Kangchenjunga (8586 m), the world's third highest peak (Figure 1). On 21 July, 1997, it was declared a conservation area spanning 2035 km^2^. The climate is alpine and harsh in the upper parts, above 3000 m, and below this elevation, mixed temperate and sub‐alpine climate is prevalent. The lower part of the study area receives snowfall for a few months and the upper range, heavy snow in winter every year. The topography is steep and rugged with mountainous terrain. The major fauna in the KCA includes snow leopards, blue sheep, wolf *Canis lupus*, red fox *Vulpes*, pikas *Ochotona* spp., common leopard *Panthera pardus*, clouded leopard *Neofelis nebulosa*, red panda *Ailurus fulgens*, musk deer *Moschus fuscus*, and Assamese monkey *Macaca assamensis* including many others (KCA, 2019; Thapa et al., 2013).

The study area comprises the headwaters of the Tamor River system and is characterized by rugged topography, with deep river canyons cutting through the lower reaches. Here, rough boulder and scree‐dominated hillsides intersperse alpine grasslands and meadows. Because of the abundant pasturage in the region, local people of upper Tamor have been practicing their livestock rearing for more than 150 years (Hooker, 1854). Yak, dzo, and cattle are common livestock kept for rearing in the area. Unlike western Nepal, domesticated small livestock are almost nonexistent, especially within snow leopard habitat.

### 2.2 Survey design for snow leopard DNA sampling

The study area was selected based on the potential habitat of snow leopards, as outlined by WWF Nepal in 2009 (DNPWC, 2017). The altitudinal range of the study was chosen between 3000 and 5500 m (using a 1:50,000 topographic map), consistent with the range both snow leopards and blue sheep have been detected across the Nepal Himalaya (Jackson & Hunter, 1996).

The target study area included all suitable snow leopard habitats within the KCA. However, due to the immensity of this area, the steep terrain and numerous cliffs, it was not possible to cover all suitable habitats. Snow leopards are difficult to detect directly and are a wide‐ranging species with an estimated annual home range of 1031.6 km^2^ for males and 470 km^2^ for females in the KCA (KCA, 2019). These estimates are significantly higher than home range estimates in other regions of Nepal such as the Longu valley and Manang (12–39 km^2^: Jackson & Ahlborn, 1989; Oli, 1997) but comparable with Mongolia for females (up to 500 km^2^: Johansson et al., 2016; McCarthy et al., 2005). Because of the high variation in home range estimates, numerous sampling grids were used to divide up (each cell: 4 × 4 km; 16 km^2^; Figure 1) potential snow leopard habitats within the KCA. A relatively large survey cell was used to allow for areas that might be inaccessible. Multi‐stage sampling (Janečka et al., 2011) was employed to ensure high interspersion of primary grids and more precise sampling cells (transects). Transects (following trail segment) were distributed within an altitudinal range of 4195–4928 m in sites that were most likely to be visited by snow leopards, especially narrow ridgelines, cliff bases, passes, and the valley bases at or immediately adjacent to frequently scent‐sprayed rocks and scrape sites.

### 2.3 Snow leopard scat collection

From early April to mid‐May (42 days) in 2012, 88 putative snow leopard scat samples were collected within 27 grids (432 km^2^) along the 140 transects (290 km). At least two transects each 1–1.5 km apart within each sampling grid was used following the technique used by Thapa (2007) adapted from Jackson and Hunter (1996). The length of transects was dependent on the terrain and human accessibility. To minimize collection of erroneous samples, scats were collected at snow leopard scrape sites and further discriminated by size and shape. A portion (~1 cm^2^; Janečka et al., 2011) of the collected scat samples were stored in 15‐ml vials with silica desiccant **(**Janecka et al., 2008), labeled (with grid number, transect number, date, location, sample ID, GPS location, etc.) and kept at room temperature prior to DNA analyses. Remaining portions of each sample were sun‐dried and put into paper envelopes for diet analysis.

### 2.4 Molecular genetic analysis

The collected samples were processed for identification of species, sex, and individual. Genetic analyses (DNA extraction, species identification, sex identification, and individual identification) of putative snow leopard scat samples were conducted following the protocol developed by Karmacharya et al. (2011), adopted from Janecka et al. (2008).

### 2.5 DNA extraction

DNA from putative snow leopard scat samples was extracted using the QiagenQIAamp DNA Stool kit following the kit protocol (Qiagen). Approximately 200 mg of silica desiccant‐preserved scat sample was surface‐scraped with sterile scissors and lysed in the lysis buffer ASL. DNA was eluted to a total volume of 150 µl. Two snow leopard samples (male and female) were included for DNA extraction from the Center for Molecular Dynamics in Nepal (CMDN) as positive controls. Negative controls were included in fecal extraction and subsequent PCR reactions to monitor contamination and false‐positive results.

### 2.6 Species identification

For snow leopard identification from fecal DNA, we amplified a 150‐bp region of the cytochrome B gene of the mitochondrial genome using primers designed by Janecka et al. (2008) and following the methods of Aryal et al. (2014). A 7 µl PCR reaction was prepared containing 3.5 µl of 5× Qiagen Master Mix, 0.7 µl of 5× Qiagen Solution, 0.07 µl of each primer, and 1.16 µl of distilled water to which 1.5 µl of extracted undiluted DNA was added. The PCR reaction was carried out at the following thermo‐cycling conditions: 95°C for 15 min, followed by 50 cycles of 94°C for 30 s, 60°C for 15 s, and 72°C for 1 min. Each assay included a known snow leopard DNA‐positive control and a “no DNA template”‐negative control.

CYTB‐SCT‐PUN F′: 5′ TGGCTGAATTATCCGATACC 3′
CYTB‐SCT‐PUN R′: 5′ AGCCATGACTGCGAGCAATA 3′

PCR products and controls (positive control using a known snow leopard sample) were visualized on a 1.8% agarose gel. The PCR of each scat sample was performed twice, and any result that was based on a single positive was repeated for a third time. Only two matching positives were considered positive snow leopard identification and used in subsequent analyses.

### 2.7 Sex identification

Sex was identified for positive snow leopard samples using felid sex‐specific primers that amplify an intron of the AMELY gene only found on the Y chromosome (AMELY F and AMELY R; Murphy et al., 1999) and following the methods of Aryal et al. (2014).

AMELY F: 5′ CCCAGCACACTCCTATTTGG 3′
AMELY R: 5′ GGAATTTCAGCTGCAAAGGA 3′

A 7 µl PCR reaction was prepared containing 3.5 µl of 2× Qiagen Master Mix, 0.7 µl of 5× Qiagen Solution, 0.07 µl of each primer, and 1.16 µl of distilled water to which 1.5 µl of extracted undiluted DNA was added. PCR thermo‐cycling condition: 95°C for 15 min, followed by 50 cycles of each 94°C for 15 s, 55°C for 30 s, and 72°C for 1 min. Each sample was run in triplicate, and the PCR products, with incorporated male and female snow leopard positive controls, were visualized on a 2% agarose gel.

### 2.8 Individual identification

Six microsatellite loci located on six different chromosomes of the snow leopard were targeted using a multiplex PCR assay, capillary electrophoresis, and genotyping. Six polymorphic microsatellite loci gave a sufficiently low probability of sampling identical genotypes based on two randomly chosen individuals. Genotyping for individual identification was optimized for multiplex PCR to allow for simultaneous amplification of several loci in one PCR reaction. We used a multiplex PCR for the following sets of loci: set 1 (PUN124, PUN229, and PUN1157) and set 2 (PUN132, PUN894, and PUN935). For each locus, one of the primers within each pair was labeled fluorescently at the 5′end to allow for visualization of alleles on an automated sequencer. Each PCR had a final volume of 7 µl and included 3.5 µl of Qiagen Master Mix (2×; Qiagen), 0.7 µl of Q‐solution (5×; Qiagen), primers for PUN124 and PUN132 at 0.5 µM, primers for PUN229 and PUN894 at 0.4 µM, primers for PUN1157 and PUN935 at 0.2 µM, and using 1.5 µl of DNA. The PCR reaction was carried out under the following thermo‐cycling conditions: 95°C for 15 min, followed by 40 cycles of each 94°C for 30 s, 55°C for 90 s, and 72°C for 90 s, and a final extension of 72°C for 10 min.

Fluorescently labeled PCR products were resolved on an ABI310 Genetic Analyzer (Applied Biosystems) automated sequencer. Allele sizes in base pairs were determined using a GeneScan 500‐Liz DNA size standard (Applied Biosystems) run in each lane. The PCR amplification and genotyping of each locus for each sample was done in triplicate. We included a positive snow leopard sample with each run to ensure alleles were sized consistently and ran negative controls with each analysis.

The size of alleles was determined using the software Genemapper 4.0. General descriptions of genetic diversity (i.e., percentage of polymorphic loci and heterozygosity) and deviations from the Hardy–Weinberg equilibrium were estimated in GenAlEx 6.41. In addition, because there were no independent samples available to estimate population allele frequencies, unique composite genotypes were used to estimate allele frequencies for P_ID_ and P_SIBS_ (the probability that siblings share a genotype). Individuals were identified using unique composite genotypes and then spatial distribution examined.

### 2.9 Population abundance and density estimation

A free software program SPACECAP package version 3.0.2 (Gopalaswamy et al., 2012) was run to estimate snow leopard density from genetic samples—a user‐friendly software package, implementing a Bayesian spatially explicit capture–recapture (SECR) analysis (Royle et al., 2009). Efford et al. (2009) recommend this software for spatial CR data originating from different types of noninvasive sources, such as camera traps, hair snares, or fecal DNA samples. SECR models in the SPACECAP package directly estimates animal density by explicitly using information on capture histories in combination with spatial locations of captures under a Bayesian modeling framework. This approach offers advantages such as substantially dealing with problems posed by individual heterogeneity in capture probabilities in conventional capture‐recapture analyses. It also offers nonasymptotic inferences, which are more appropriate for small samples of capture data typical of photo‐capture studies.

A detailed discussion of SECR analysis is beyond the scope of this study. The reader is referred to Royle et al. (2009), cited before for more detailed discussion of further technical details about the models and analyses. However, the major assumptions of SECR analysis are (1) population closure (no exist or entry into the population through mortality and recruitment from the study area, (2) individual activity centers are randomly distributed and do not change for the duration of study, (3) each animal has an activity center and the probability of capture decreases with distance to that activity center, and (4) there is independence in individual encounters among individuals and within the same individuals. Our sample collection duration was over a short period (42 days), which was well‐tied with Assumption 1. We used a pixel size of 1 km × 1 km to estimate the density distribution of activity centers of individual snow leopard ranges. We excluded permanent snow and water bodies, agricultural land and extensive forest patches, and areas below 3000 and above 5500 m altitude (DNPWC, 2017). We assumed that these excluded areas were not preferred habitat for snow leopards. For detection probability, we configurated the expected encounter rate at distance 0 from the activity center (λ0). We examined the effect of topography and altitude on the encounter rate at the activity center, and we expected that the average ranging parameter was well‐fitted across the study area and sample size of encountered snow leopards. For model selection to examine the relationship between snow leopard density, detection, and explanatory variables, we utilized the Akaike information criteria (AIC; Burnham & Anderson, 2002).

Following Singh et al. (2010), three types of input files were prepared to analyze data in the SPACECAP package: (i) animal capture details, (ii) trap deployment details, and (iii) state‐space details. The trap deployment file consisted of trap location ID and spatial location of trap IDs in *X* and *Y* coordinates (Universal Transverse Mercator UTM projection system) along with information on the occasions when each individual location was operational during the sample collection duration. Here, the mid‐center of grids was used as detectors (location), and we reduced the number of sampling occasions by pooling the data together across three days due to a low encounter probability, as suggested by Janečka et al. (2011). The trap deployment data were organized in a two‐dimensional matrix of individually identified locations and sampling occasions in a binary format (1’s and 0’s), indicating whether an individual location was or was not operational on a particular sampling occasion. A mesh of points (potential home range center) in each cell (0.25 km^2^) was generated using the Arc Geographical Information System (GIS) in a sampling array and 1.6‐km buffer. The potential home range center file consisted of *X* and *Y* coordinates of all the potential activity centers in the UTM Projection System, and a habitat suitability indicator (using 1’s or 0’s) representing the potential activity centers within suitable or unsuitable habitat. The analysis was run in SPACECAP v 3.1 under the program R environment. For MCMC simulation, 60,000 iterations were selected, 1000 burn‐in values (number of initial values to discard during the MCMC analysis), and 1 as thinning, which was recommended by Royle et al. (2009). Only iteration numbers defined by the thinning rate are stored for the analysis (Singh et al., 2010). As there was uncertainty about the total number of animals, which was likely to be larger than the minimum number identified during fecal DNA analysis, the value needed to be “augmented.” Here, fecal DNA analysis identified 19 individual snow leopards in this survey; thus, data augmentation has been set at 100 as a rule thumb; this was 5 times greater (Royle et al., 2009).

### 2.10 Subsistence prey: blue sheep and livestock

Based on local knowledge and alleged dispersal barriers such as high peaks, deep gorges, and glaciers, we surveyed blue sheep and livestock within potential snow leopard habitats in the KCA. Blue sheep survey blocks were selected as follows:

1. Gola block comprising the western border of Walungchung Gola and extending up to Tiptala in northern China, to the Nup Himalayan range in the east.
2. Yagma block comprising the Nup Himalayan range along with Khangla along the northern border of China, to Nanggola in the east.
3. Khambachen block extending from Nanggola to the Jhonu Himalayan range in the east.
4. Ramjer block extending from the Jhonu Himalayan range to Ratong La and Khang La along the eastern border of India.

In spring of 2012, we performed direct counts of blue sheep from appropriate vantage points (also known as a fixed‐point count) within the four blocks of the KCA. The total number of individuals in a group was counted and classified by sex and age. To classify the sex and ages of blue sheep, a Snow Leopard Monitoring Guideline (Thapa, 2007) was utilized (adopted from Wegge, 1979). Observations were made with 8–24× binoculars and 15–60× spotting scope early in the morning (06.00 am−10.00 am) and afternoon (14.00 pm–17.30 pm) at Nepal Standard Time (NST) when blue sheep are known to be most active. Blue sheep densities were estimated by dividing the total number of animals observed by the total area sampled within four blocks. The sampled area was obtained by adding up areas of all surveyed blocks. The area of each block was obtained by demarcating them on a GIS platform (i.e., Google Earth program) post‐survey by the team of surveyors.

For domestic livestock information, opportunistic counting of livestock numbers was made in the pasture and consultation with key informants were used to verify the data obtained during the study.

### 2.11 Snow leopard diet

From known snow leopard scat samples derived from DNA analysis, a total of 73 collected scat samples were dried in the shade, labeled with a unique code, and stored in an envelope for laboratory analyses. The samples were washed with tap water through a fine mesh sieve (1 mm) and oven‐dried at a temperature of approximately 60°C. Each sample was further cleaned in an ethanol–alcohol mixture (1:1). Food items from scat samples were identified mainly from the macro‐ and microscopic structures of hairs. Remains such as bones, teeth, hooves, feathers, plants, and stones were noted but not included in the analysis of diet. We randomly selected 20 strands of hair from each scat sample and mounted them on microscope slides. A gelatin solution was used to prepare slides to show the cuticular structure of hairs and examine the medulla structure and cross section of the hair; cuticular scales were observed by the impression technique (Oli, 1993). To identify hair samples, we compared them with reference samples from potential prey species (9 wild and 6 domestic stock) around the study site using microscopic images from a model B K Biological Trinocular Microscope (10×, 100×, 400× and 1000×) and a DCM510 digital camera following the methods describe in Oli (1993). The cuticular scale patterns of hairs were analyzed and further validated by medullary structure and cross section (Joslin, 1973; Koppikar & Sabnis, 1976). We did diet analysis at Tribhuvan University laboratory, and subsequently, it was cross‐validated by a laboratory technician. However, we did not estimate human error for this purpose. Results were expressed as the relative frequency of prey species found in the samples.

#### 2.11.1 Conversion of prey biomass

An estimate of biomass consumed by snow leopards was used, rather than occurrence percentage, as diet quantification may be more reliable (Wegge et al., 2012). Smaller animals, for example, are usually overrepresented in the scat analysis (Floyd et al., 1978). The frequency of occurrence of individual prey items is converted to biomass and numbers consumed according to the relationship between scat production and prey size (Ackerman et al., 1984; Floyd et al., 1978) in the following way: *Y* = 1.980 + 0.035 *X*, where *Y* is weight of prey consumed per scat and *X* is the live weight of particular prey (in kg).

Earlier studies of wolves (Floyd et al., 1978) and cougars *Felis concolor* (Ackerman et al., 1984) developed regression equations based on captive feeding trials using prey of different body sizes. Derived from experimental feeding trials on cougar and widely used in diet studies of snow leopards and other felids (Wegge et al., 2012; and reference therein), we used this equation to estimate the relative weights consumed of different prey species in the diet.

To quantify the predation rate of a snow leopard, average consumption rates per day of wild prey (blue sheep) and domestic livestock were used following Oli et al. (1993) and Wegge et al. (2012) as follows: blue sheep 34 kg, Yak 150 kg, horse/cattle 140 kg, goat 25 kg, and sheep 30 kg. Of large prey, predators did not consume the whole ungulate carcass due to inedible parts such as skeletal pieces, horns/antlers, rumen content, and parts of the skin (Floyd et al., 1978). It was assumed that snow leopards consumed the following proportional weights of domestic and wild ungulate, as described by Oli et al. (1993) and Wegge et al. (2012): blue sheep 75%, yak 50%, horse and cattle 60%, and sheep and goat 70%.

A G‐Test was employed to determine if snow leopards have a preference species according to their availability across the study sites (Manly et al., 2007). To determine if particular species will be preferred, less preferred or avoided, a 95% confidence interval was created for each category by applying Bonferroni corrections to the *Z*‐statistic (Neu et al., 1974).

## 3 RESULTS

### 3.1 Snow leopard abundance

**FIGURE 2:**
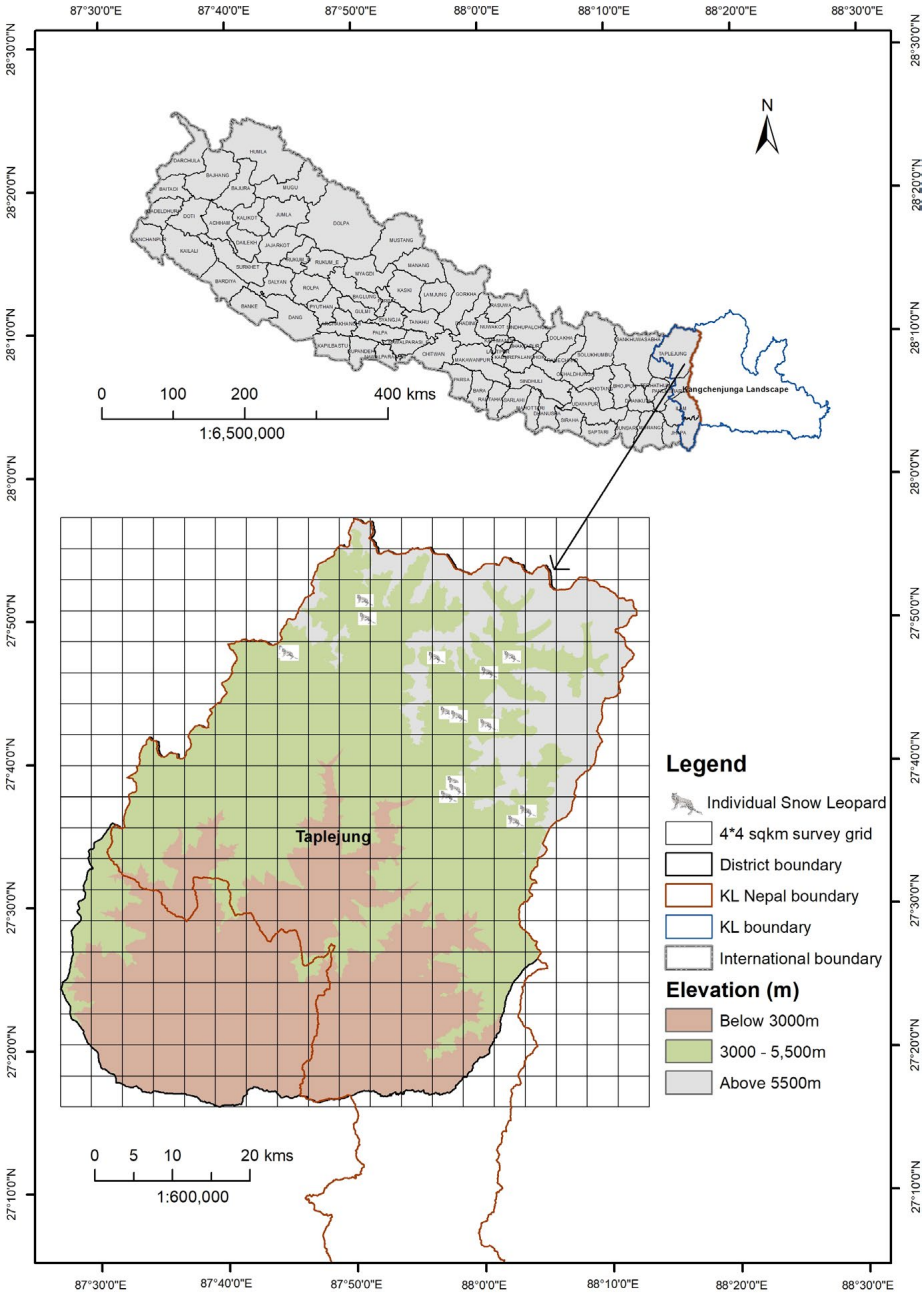
Location points of individual snow leopard identified by fecal DNA analysis within the potential snow leopard habitat in the KCA during the Spring Season (April–May) 2012

Out of 88 putative fecal samples, 73 (83%) were confirmed by genetic analyses to be from snow leopards. Among snow leopard‐positive samples, 60% (44) were successfully genotyped. Fecal DNA analysis identified 19 individual snow leopards (nine females and ten males; Table 1; Figure 2). The program SPACECAP estimated a mean snow leopard population size of 24 (95% CI: 19–29; SD: 3.04). The mean density was 3.9 individuals/100 km^2^ (95% CI: 3.1– 4.8; SD: 0.8; Table 1) for the survey area of 609 km^2^. The Khambachen block had a higher number of snow leopards, followed by Ramjer (eastern part of Khambachen), Yagma, and Gola (both blocks lie northwestern part of Khambachen).

**TABLE 1:** Summary of model parameters for snow leopard fecal DNA analysis from the KCA, 2012

Item	Mean	SD	CI 95% (lower level)	CI 95% (upper level)
Sigma	1.73	3.23	0.21	1.19
lam0	0.05	0.09	0.03	0.06
Psi	0.08	0.02	0.04	0.11
Nsuper	23.57	3.04	19	29
Density	3.9	0.8	3.1	4.8

Note

(Bayesian *p*‐value based on individual encounters: .75); sigma: encounter probability, lamda: detection probability at trap location that considered home range, psi: data augmentation parameter (the ratio of the number of animals actually presents within S to the maximum allowable number), Nsuper: population size, and density: animals per 100 km^2^).

The encounter probability was 1.73 (95% CI: 0.21–1.19). Detection probability at trap locations was found to be 0.05, and the psi, the ratio of the number of animals actually present within the space to the maximum allowable number, was 0.08. The number of effective alleles (A_e_) was 2.4 (SE ± 0.18), and observed heterozygosity (H_o_) was 0.57 (SE ± 0.08) in six polymorphic loci.

### 3.2 Available biomass of blue sheep and livestock

Within the four blocks of the study area, a total of 45 blue sheep groups with 1404 individuals were counted and the estimated density was 2.3/km^2^ (Table 2) in 609 km^2^. Recruitment was estimated to be 44 young per 100 adult females in the spring season. The mortality rate of yearlings was estimated to be 45%, which could be consistent with a similar recruitment number in the previous year. Overall, the sex ratio was female‐biased (73 male per 100 adult female).

**TABLE 2:** Demographic population structure of blue sheep in the KCA, 2012

Block	Group	FE	YO	YE	YM	MM	BM	UI	Total
Ramjer	4	42	20	7	10	5	5	15	104
Khambachen	17	267	99	37	37	78	89	27	634
Yagma	16	177	82	64	50	43	41	53	510
Gola	8	54	34	21	15	13	8	11	156
Total	45	540	235	129	112	139	143	106	1404

Note

Table describes sex and age of blue sheep within each sampling block. The group category gives the total number of blue sheep individuals within the sampling block, FE = the number of adult females (more than 2 years), YO = the number of young (below one year age of both male and female), YE = the number of yearlings (in between one and two year ages of both male and female), YM = the number of young males (above two and below four years), MM = the number of middle males (above four years and below seven years), BM = the number of big males (above seven years), and UI = the number of those unidentified (all ages both male and female) Thapa (2007) adopted from Wegge (1979).

In the KCA, yaks (dzo, dee, jhomo) dominated livestock types, but small livestock such as goat and sheep are negligible in this region. The total estimated available standing biomass of yak was over six times greater than that of blue sheep (Table 3). The total number of yak (2050) and blue sheep (1404) together accounted for 355,236 kg (yak: 307,500 kg, i.e., 505kg/km^2^ and blue sheep: 47,736 kg, i.e., 78 kg/km^2^) available standing biomass in the KCA.

**TABLE 3:** Available standing biomass of yak and blue sheep in the KCA, 2012

Block	No. of yak	Biomass (kg)	No. of blue sheep	Biomass (kg)
Gola	459	68,850	156	5304
Yagma	601	90,150	510	17,340
Khambachen	804	120,600	634	21,556
Ramjer	186	27,900	104	3536
Total	2050	307,500	1404	47,736

Note

We assume an average weight of 150 kg for a yak and an average weight of 34 kg for a blue sheep (adopted from Wegge et al., 2012).

### 3.3 Snow leopard diet

Out of 73 snow leopard scat samples, micro‐histological analysis revealed that one third of scats consisted of multiple prey and two thirds of single prey species. The prey consumed included three wild and four domestic mammals. Wild prey accounted for 49%, whereas domestic prey accounted for 45%, with the remainder unidentified, 6% (SD = 11.2; *N* = 8; Table 4). Blue sheep dominated wild prey in the snow leopard diet (70%; SD = 3.1 among total available wild prey species, *N* = 3), and yak was the dominant domestic prey item, accounting for approximately 43% of the total domestic livestock in the snow leopard diet (SD = 7.02, *N* = 4).

**TABLE 4:** Count and frequency of prey items in the snow leopard diet from microscopic analysis of fecal material in the KCA (*n* = 73), 2012

Prey species	Count	Frequency of occurrence
Blue sheep	70	34.3
Musk deer	11	5.4
Pika	19	9.3
Yak	40	20
Cow	7	3
Goat	35	17
Sheep	10	5
Unidentified	12	6
Total	204	100

The harvested biomass by one snow leopard per day was estimated at 3.7 kg/day (Table 5). This is equivalent to 1343 kg annually. This annual biomass estimate, in the KCA, would amount to 21 blue sheep, 2 musk deer, 318 pika, 2 yak, 0.5 cattle, 4 goats, 0.3 sheep, and other small mammals including birds (Table 5). Snow leopards preferred blue sheep to yaks, goats, and sheep, whereas they avoided cows and musk deer on prey biomass availability (Table 6).

**TABLE 5:** Estimation of prey species consumed in a year by a snow leopard in the KCA, 2012

Prey species	Estimated mass (A)	Biomass/ scat (B) correction factor	Frequency of occurrence (C)	Biomass consumed (D)	Biomass eaten (E)	No. of prey consumed by a snow leopard (F)
Blue sheep	34	3.17	70	221.9	710.08	21
Musk deer	16.5	2.56	3	7.67	24.55	2
Pika	0.3	1.99	15	29.86	95.54	318
Yak	150	7.23	10	72.3	231.36	2
Cow	140	6.88	3	20.64	66.05	0.5
Goat	25	2.86	11	31.41	100.50	4
Sheep	30	3.03	1	3.03	9.70	0.3
Others	2	2.05	16	32.8	104.96	52
Biomass (KG) consumed by a snow leopard/year				420	1343	

Note

A = assumed weight of the prey species; B = Y (i.e., Y = 1.98 + 0.035*A); C = frequency of occurrence in scats; D = biomass consumed (B*C); E = biomass eaten by a snow leopard assuming a rate of 3.7 kg/day; F = no. of prey species consumed by a snow leopard/ year based on E divided by A.

**TABLE 6:** Utilization, availability, and preference of prey species in snow leopard diet within the KCA (2012)

Species	Utilization		Availability	90% Bonferroni confidence interval		Preference
	Counts	Proportion	Proportion	Lower	Upper	
Blue sheep	70	0.40	0.07	0.38	0.44	+
Musk deer	11	0.05	0.06	0.02	0.04	−
Pika	19	0.08				NA
Yak	40	0.18	0.52	0.25	0.30	0
Cow	7	0.03	0.06	0.03	0.05	−
Goat	35	0.16	0.13	0.08	0.11	0
Sheep	10	0.04	0.03	0.02	0.04	0

Note

Symbols (−, 0, +) denote prey species that are avoided, less preferred, and more preferred respectively, according to their availability (based on 95%, Bonferroni confidence intervals).

### 3.4 Snow leopard prey availability

An annual food requirement for one snow leopard is 1343 kg. The standing available food biomass of both wild and domestic livestock was calculated at 355, 236 kg. Blue sheep, the snow leopard's staple food in the KCA, was calculated at 47, 736 kg and domestic yak, 307, 500 kg. The harvesting rate of the estimated 24 snow leopards was about 7% per annum. If a snow leopards killed 21 blue sheep annually, then the extant 24 snow leopards would require 504 blue sheep per annum. Without the biomass contribution of small mammals and livestock, the estimated available blue sheep biomass of 47, 736 kg gives a predator‐to‐prey ratio of 1:59 in the KCA on a weight basis. According to our blue sheep count data, the spring population in the KCA consisted of 17% young (kids) and the annual predation rate was estimated at 28% of the blue sheep population.

## 4 DISCUSSION

Our estimates of snow leopard abundance, diet composition, and wild prey availability suggest that available wild prey biomass is insufficient to support the estimated snow leopard population in the KCA of eastern Nepal, without supplementation of domestic livestock. In the KCA, we estimated 24 snow leopards would need to consume 480–720 blue sheep per year to survive. Our estimates are consistent with Jackson and Ahlborn’s (1989) hypothesis that an adult snow leopard requires 20–30 blue sheep annually with 150–230 blue sheep available (possibly, less in areas where other wild prey species are easily available). The snow leopard‐to‐blue sheep ratio in the KCA is considerably lower than this required availability (1:59), indicating insufficient wild prey to sustain snow leopard populations in the area. Lovari and Mishra (2016) suggested that a declining wild ungulate population would ultimately threaten snow leopards in the long run. In the central region of Manang, the ratio is substantially higher and sufficient (according to the above hypothesis) for snow leopard survival (1:114–1:159; Oli, 1994). In the KCA, blue sheep are the only optimal sized wild prey available for snow leopards, and other important natural prey species such as marmots *Marmota himalayana*, and woolly hare *Lepus oiostolus* are absent (Thapa, 2006), preferring drier habitats. Only pika constitutes suboptimal prey in the area.

Why the considerable difference in the snow leopard‐to‐blue sheep ratio between the KCA in the east and Manang in the center? One possibility is that blue sheep populations in the KCA may be indirectly impacted by the prevalence of large livestock such as yaks, pushing snow leopards to target young blue sheep as they are smaller and easier to capture. In the KCA snow leopards were estimated to harvest 28% of young blue sheep of the total blue sheep population annually, nearly twice the amount estimated (15.1%) by Wegge et al. (2012) in the Phu valley (Manang). The proportion of young blue sheep in the snow leopard prey base is similar between the KCA and Phu (17% vs. 18.4%; Wegge et al., 2012). With less small livestock available in the KCA compared with western and central regions (Dolpa, Manang and the Phu Valley; Devkota et al., 2013; Oli, 1994; Wegge et al., 2012), snow leopards may have targeted young blue sheep instead, likely suppressing the greater blue sheep population in turn. Lovari et al. (2009) reported that in Mt. Everest, also of eastern Nepal, in the absence of domestic goats and other small prey, the Himalayan tahr young‐to‐female ratio was substantially decreased (0.8–0.9 in early summer to 0.1–0.2 in autumn) by snow leopard predation, eventually altering the population dynamics by removing an entire age class. In the KCA, snow leopards share habitat with two other apex predators, the common leopard along the forest edge habitat in the south (Thapa et al., 2013) and the grey wolf (Subba et al., 2017) in the north, which would add predation pressures on blue sheep populations.

At a certain blue sheep low abundance threshold, snow leopard prey preference in the KCA appears to have switched to livestock. The available prey biomass of blue sheep and snow leopard diet analysis suggest that snow leopards in the KCA are being pushed to target even large livestock such as yaks and actually depend on it for survival, despite the lack of preferred small livestock prey. Our total blue sheep available biomass in the KCA was only 78kg/km^2^ (2.3/km^2^). Khorozyan et al. (2015) in their logistic regression analysis of recent peer‐reviewed scientific publications showed that cattle predation is high when wild prey biomass is below 812.41 ± 1.26 kg/km^2^, whereas domestic sheep and goat predation is high at less than 544.57 ± 1.19 kg/km^2^, regardless of sizes of areas of study and species, body masses, and population densities of cats. With such a low biomass of blue sheep available in the KCA, it is therefore not surprising that snow leopards are forced to target yaks that are much more available (biomass = 505 kg yak/km^2^) and the only available livestock in the region. The vast majority of animals killed by snow leopards are smaller than 100 kg (Lovari, Ventimiglia, & Minder, 2013, Lovari, Minder, et al., 2013), so these are likely to be young yaks. Furthermore, despite not being able to identify the ages of yaks from our snow leopard dietary analysis, in a social survey of the community, 100% of respondents reported losing yak calves (1–2 year ages) over the last one to two years, and not adult yak. This finding is consistent with our dietary study that revealed a higher proportion of livestock in snow leopard diet within the KCA (45% livestock and 49% wild prey) than in other regions in Nepal, with yaks being the dominant prey source (43%). In most other regions, livestock were not nearly as prominent in the snow leopard diet—on central Nepal: Annapurna–Manaslu region (57% blue sheep and 31% livestock; Chetri et al., 2017), Annapurna region: Manang and upper Mustang (63% blue sheep and 18% livestock; Aryal et al., 2014); in western Nepal: SPNP (70% wild prey and 30% livestock; Devkota et al., 2013); and in eastern Nepal: Everest region (56% Himalayan tahr and 25% livestock; Ferretti et al., 2014; Lovari, Ventimiglia, & Minder, 2013, Lovari, Minder, et al., 2013). In all these regions, snow leopard prey preference appears to be influenced by a greater available biomass of wild prey than the KCA (e.g., only 78 kg/km^2^ in the KCA compared with a range of 200–304 kg/km^2^ in other regions). However, in the Phu valley of central Nepal, wild prey (blue sheep) are abundant (Wegge et al., 2012) and small livestock, which are also plentiful, still accounted for 42% of snow leopard diet, so available easy prey are also likely an influencing factor. In India, Bagchi and Mishra (2006) found that high livestock depredation (58% of snow leopard diet) coincided with high livestock densities (29.7 heads/km^2^) and a shortage of wild ungulate prey (2.1–3.1 blue sheep/km^2^) in contrast to an adjoining area where livestock represented 40% of snow leopard diet with a reduced livestock density (13.9 heads/km^2^) and greater numbers of available wild prey (4.5–7.8 ibex/km^2^). On average globally, livestock constitute 23.5% of the total prey biomass consumed by snow leopards (Lyngdoh et al., 2014). Jackson (2015) also concluded from the revision of several snow leopard dietary studies that livestock, on average, comprise between 15% and 30% of snow leopard diet. It is clear from snow leopard dietary studies across Nepal and the other range countries that livestock play an important role in snow leopard survival (Johansson et al., 2015; Mishra et al., 2016; Oli et al., 1993; Sharma et al., 2015; Shehzad et al., 2012; Shrestha et al., 2018; Wegge et al., 2012), but in the KCA, with such low wild prey availability, it may be an even more crucial element for the snow leopard survival.

Given the already low abundance of blue sheep numbers in the KCA, 7 times lower than the predicted threshold (Khorozyan et al., 2015), if blue sheep abundance continue to decline, snow leopards will further depend on livestock, and obviously the risk of retaliatory killings of snow leopard will increase, as has been noted in other parts of the Himalayas (Ale et al., 2007; Schaller, 1998). Nowell et al., 2016 reported that 55% of snow leopard killings by humans are a result of retaliation and are increasing. The general assumption in the big cat world is that an increase in wild prey availability reduces livestock depredation. The revival of wild prey populations may at least partially mitigate livestock depredation through effective conservation intervention (Bagchi et al., 2019). In the Mt. Everest region of Nepal, the recovery of wild ungulate populations helped in the initial recolonization of snow leopards (Ale et al., 2007). However, to increase blue sheep abundance 7‐fold to offset livestock killing in the KCA, as suggested by Khorozyan et al. (2015), is a highly improbable management achievement, at least in the short‐term. The literature on snow leopard conservation suggests that management solutions are instead, not so clear‐cut, with cause‐and‐effect patterns being a lot more intricate. McCarthy and Chapron (2003) suggested that the importance of each contributing factor to snow leopard livestock depredation varies across landscapes and between countries, even at site‐specific levels, thus requiring site‐specific conservation action. There is also a fine balance in finding the optimal threshold of wild prey and predator abundance. If wild prey increases enough to produce a significant increase in snow leopard numbers, snow leopards may target livestock more due to an increased encounter rate, as suggested by Suryawanshi et al. (2013). There is also the fact that small livestock including goats may be more appealing to snow leopards, despite adequate wild prey populations. Therefore, conservation initiatives aimed at recovering wild prey populations should also be accompanied by greater measures to protect livestock and compensating economic damage caused by carnivores (Suryawanshi et al., 2017). Krafte Holland et al. (2018) through a systematic review of peer‐reviewed literature on human–big cat conflict and potential solutions found that compensation schemes and livestock management strategies were more effective for addressing conflict than other interventions. Given this finding and our results, snow leopards in the KCA region of Nepal would benefit from a multi‐pronged conservation management approach: (1) the supplementation of wild prey, (2) education of locally impacted communities, (3) increased protection of livestock, and (4) financial compensation strategies for the loss of livestock. However, knowing exactly how much wild prey is needed to attain ecological balance will require the statistical modeling of different factors including snow leopard abundance, availability of domestic prey, size of domestic prey, husbandry practices, and their level of protection. In addition to this, with humans increasingly encroaching on snow leopard habitats, should domestic livestock play a role in snow leopard conservation? In most of the snow leopard's range, current community conservation initiatives do focus on compensation and incentive, rather than preventative measures. Recently introduced community‐managed livestock insurance schemes in the KCA are offering huge promise for the long‐term survival of snow leopards by reducing the incentive for retribution killings (Gurung et al., 2011). Along with improvements to this scheme (e.g., more appropriate compensation percentages and linkages with government policy), there needs to be a focus on engaging and educating local people to be citizen scientists on the importance of snow leopard conservation, involving them in long‐term monitoring programs and promotion of ecotourism. The solution to big cat long‐term conservation is never simple, but interdisciplinary collaboration and a multi‐pronged approach will likely provide the ultimate key to this paramount issue.

## CONFLICTS OF INTEREST

None.

## AUTHOR CONTRIBUTION

**Kamal Thapa:** Conceptualization (equal); Data curation (lead); Formal analysis (equal); Funding acquisition (lead); Methodology (equal); Writing‐original draft (lead); Writing‐review & editing (equal). **Natalie Schmitt:** Writing‐review & editing (equal). **Narendra M. B Pradhan:** Conceptualization (equal); Supervision (lead); Writing‐review & editing (supporting). **Hem Raj Acharya:** Conceptualization (supporting); Data curation (supporting); Writing‐original draft (supporting). **Santosh Rayamajhi:** Conceptualization (equal); Supervision (equal); Writing‐review & editing (supporting).

## Data Availability

The scat sample locations, DNA sequences, and microsatellite genotypes are available at the Dryad Digital Repository https://doi.org/10.22541/au.161191408.89015908/v2
