# Correction to “No silver bullet? Snow leopard prey selection in Mt. Kangchenjunga, Nepal”

**DOI:** 10.1002/ece3.70138

**Published:** 2024-08-06

Thapa, K., Schmitt, N., Pradhan, N. M. B., Acharya, H. R., & Rayamajhi, S. (2021). No silver bullet? Snow leopard prey selection in Mt. Kangchenjunga, Nepal. *Ecology and Evolution*, 11, 16413–16425. https://doi.org/10.1002/ece3.8279

Data were not previously uploaded in Dryad, but now can be accessed at https://doi.org/10.5061/dryad.vq83bk3rn.

We apologize for this error.

